# Vibration-Induced Alteration in Trunk Extensor Muscle Proprioception as a Model for Impaired Trunk Control in Low Back Pain

**DOI:** 10.3390/brainsci14070657

**Published:** 2024-06-28

**Authors:** John R. Gilliam, Debdyuti Mandal, Peemongkon Wattananon, Sourav Banerjee, Troy M. Herter, Sheri P. Silfies

**Affiliations:** 1 Applied Neuromechanics Lab, Department of Exercise Science, University of South Carolina, Columbia, SC 29208, USA; jrg15@email.sc.edu; 2 Integrated Material Assessment and Predictive Simulation Laboratory (i-MAPS), Department of Mechanical Engineering, University of South Carolina, Columbia, SC 29208, USA; dmandal@email.sc.edu (D.M.); banerjes@cec.sc.edu (S.B.); 3 Motor Control and Neural Plasticity Laboratory, Faculty of Physical Therapy, Mahidol University, Nakhon Pathom 73170, Thailand; peemongkon.wat@mahidol.edu; 4 Department of Exercise Science, University of South Carolina, Columbia, SC 29208, USA; hertert@mailbox.sc.edu

**Keywords:** back muscle vibration, proprioception, chronic low back pain

## Abstract

This study examined the impact of personalizing muscle vibration parameters on trunk control. We assessed how altered trunk extensor muscle (TEM) proprioception affects seated trunk control in healthy controls (HCs). To explore the link between altered TEM proprioception and impaired trunk control in chronic low back pain (cLBP), we performed equivalence testing between HCs undergoing TEM vibration and cLBP without vibration. Twenty HCs performed active joint reposition error (AJRE) testing to determine personalized vibration parameters. Each participant maintained balance on an unstable chair with eyes open and closed, with and without TEM vibration. We compared trunk control between HCs and twenty age- and sex-matched cLBP participants, using mean velocity and 95% confidence ellipse area of center-of-pressure changes to quantify trunk postural control. Equivalence was examined by comparing mean difference scores to minimal detectable change values and calculating between-group effect sizes. Personalized vibration parameters led to larger lumbopelvic repositioning errors (d = 0.89) than any single vibration frequency (d = 0.31–0.36). In healthy adults with no back pain, vision had large effects on postural control (η_p_^2^ = 0.604–0.842), but TEM vibration had no significant effects (*p* > 0.105) or interactions with vision (*p* > 0.423). Between-group effect sizes (d = 0.32–0.51) exceeded our threshold for performance equivalence (d < 0.2). Muscle vibration altered position sense during AJRE testing, and personalizing parameters amplified this effect. However, TEM vibration had minimal impact on seated trunk postural control in adults with no back pain and did not lead to performance degradation comparable to that in cLBP.

## 1. Introduction

Trunk postural control is defined as the ability to maintain the position and stability of the trunk during static and dynamic tasks. It involves the coordinated activation of muscles to stabilize the spine and pelvis. Effective trunk postural control is essential for performing everyday tasks and maintaining balance, as it provides a stable base for limb movements and helps prevent falls and injuries [1,2]. Trunk postural control is a critical component of overall postural stability and is influenced by sensory inputs (including those from the visual, vestibular, and somatosensory systems) and motor responses [2,3,4]. Altered trunk postural control has been associated with the development of low back pain [5] and the persistence of symptoms [6,7]. Trunk postural control relies on proprioceptive information from receptors in the skin, joints, tendons, and muscles to coordinate muscle activations at multiple joints. [3,4,8]. Muscle spindles are a particularly important source of proprioceptive information due to their high densities within trunk muscles [9,10]. In individuals with chronic low back pain (cLBP), trunk extensor muscles (TEMs) in the lower back exhibit characteristic structural and morphological changes, such as fatty infiltration and fibrosis [11,12,13,14,15]. These changes may compromise trunk postural control by altering the proprioceptive information relayed by muscle spindles in TEMs [16,17,18]. However, the relationships between cLBP and altered TEM proprioception remain unclear.

Vibration being applied to skeletal muscles causes increased discharge from primary muscle spindle afferents, resulting in altered proprioception and a kinesthetic illusion that the vibrated muscle is lengthening [19,20,21,22,23,24,25,26]. While these effects are observed in different muscle groups, research paradigms, and study samples, including individuals with cLBP [21,27,28,29,30], up to one-third of individuals in previous studies did not experience a noticeable effect due to vibration [18,30]. A limitation of previous trunk muscle vibration studies is the application of the same vibration parameters for all participants [17,29,31,32,33,34]. Moreover, the vibration frequencies used in previous investigations of TEMs were based on studies that elicited a response in extremity muscles [21,22]. In addition, vibrations employed in previous studies were applied using DC motors that prohibited the individualization of the vibration parameters [17,18,31,34]. Finally, muscle vibrators were typically secured to trunk muscles using belts and straps that alter the proprioceptive information received from cutaneous receptors, thereby adding a confounding signal to the contributions of the TEM spindles [17,18,31,34].

Another limitation of prior research investigating the effects of TEM vibration on trunk postural control is that most studies assessed postural control in standing [17,33,35]. While studies in standing have demonstrated impairments in proprioceptive reweighting in individuals with cLBP, assessing trunk postural control in sitting may enhance the ability to examine the effects of TEM vibration by decreasing the availability of proprioceptive information from the lower extremities that is pertinent to task performance. One previous study that examined the effects of TEM vibration on trunk postural control during sitting did not include an eyes-closed (EC) condition [34]. Eyes-open (EO) conditions lead to a greater reliance on visual feedback, which is associated with cLBP [17,36]. Accordingly, the inclusion of an EC condition allows for a more specific assessment of the effects of altered TEM proprioception on trunk postural control without compensation from the visual system.

The purpose of this study was first to determine the effect of personalizing vibration parameters by presenting individuals with multiple vibration frequencies and determining which frequency elicited the greatest error in TEM proprioception. We then used personalized vibration parameters to characterize the effects of altered TEM proprioception on seated trunk postural control in EO and EC conditions in people with no back issues. Additionally, to evaluate TEM vibration as a model for exploring the relationship between altered TEM proprioception and trunk postural control in cLBP, we compared trunk postural control between individuals with no back issues undergoing TEM vibration and persons with cLBP without vibration. We hypothesized that personalized vibration parameters would result in greater errors in proprioception than a single vibration frequency applied to all participants. We also hypothesized that healthy individuals would exhibit poorer trunk postural control under EC conditions and while undergoing TEM vibration compared to undergoing no vibration. Finally, we hypothesized that the sitting trunk postural control would be equivalent for individuals with no back issues experiencing TEM vibration and individuals with cLBP.

## 2. Materials and Methods

### 2.1. Participants

Twenty healthy adults with no history of back pain were recruited from the local community. A history of back pain was defined as missing three days of work or recreational activity or resulting in seeking care from a healthcare professional. Participants were excluded if they had a history of concussions or migraine headaches within the last six months, a history of neurological disease, previous spine or hip surgery, a diagnosis of fibromyalgia, rheumatoid arthritis, or chronic fatigue syndrome, or were currently taking opioids. The study was approved by the University of South Carolina Institutional Review Board (Pro00124463), and all participants gave their written informed consent prior to testing.

Additionally, we leveraged a previously collected dataset of 54 individuals with cLBP who were tested using an identical protocol and equipment but without muscle vibration. The study of cLBP participants was approved by the Institutional Review Board of the University of South Carolina (Pro00079198). We selected age- and sex-matched individuals with cLBP for comparison with participants with no back issues. Participants with cLBP had symptoms between the 12th rib and gluteal fold for >3 months, experienced symptoms at least half the days in the previous 3–6 months, and reported activity limitations due to their back pain [37]. Participants who experienced lower extremity symptoms related to back pain were excluded. Detailed inclusion and exclusion criteria for both participant groups can be found in the Supplementary Materials.

An a priori power calculation was performed based on within-factors effects from repeated measures ANOVA testing. We estimated a moderate effect size (f = 0.25), with alpha = 0.05, power = 80%, three measurements for each seated trunk postural control condition, and a correlation between measures of 0.7. We based our effect estimate and correlation between measures on previously collected data using the same protocol (absent vibration) and primary outcome variables (CEA_95_ and MVEL). We previously recorded large effects for healthy individuals by manipulating visual feedback when comparing eyes-open (EO) and eyes-closed (EC) conditions [36]. In previous experiments of postural control, manipulating vision has led to larger effects than vibration [34]; therefore, we expected a moderate effect when manipulating proprioception alone. Power analysis indicated that 18 participants were needed to detect the hypothesized effects.

Table 1 presents the participant demographics and data from self-report measures. To characterize the back pain intensity and the degree to which back or leg pain impacts functional activities in our 20 matched cLBP subjects, we used the Numeric Pain Rating Scale (0–10) [38,39] and the Oswestry Disability Index [40,41,42], respectively. The groups were not different regarding the participants’ sex, age, body mass index, or physical activity level. The cLBP group presented with moderate pain and relatively low disability due to back pain. One participant with no back issues was excluded from AJRE and postural control testing data due to an inability to maintain electrogoniometer contact on the back during testing.

### 2.2. Personalized Vibration Parameters

**Figure 1:**
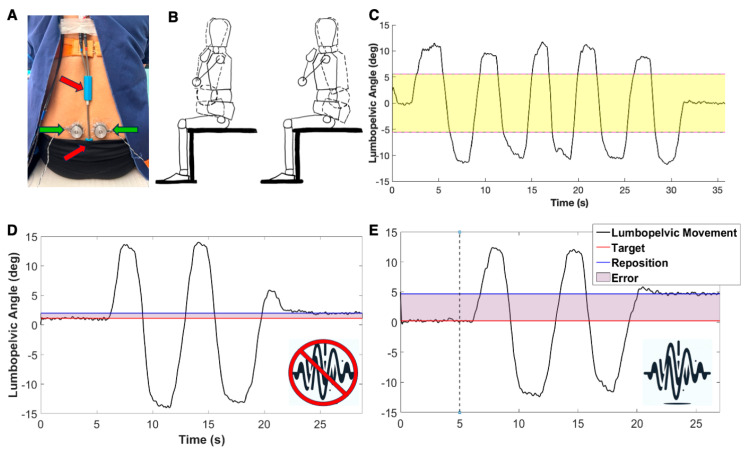
(**A**) Red arrows: the electrogoniometer with ends positioned at the sacrum (S2) and first lumbar vertebrae (L1). Green arrows: the muscle vibrators positioned two cm lateral to the fourth lumbar (L4) spinous process. (**B**) Movement was focused on sagittal plane lumbopelvic movement (anterior and posterior pelvic tilting). (**C**) Single-subject time series data of active ranges of motion, tilting the pelvis anteriorly and posteriorly five times through the full active range of motion. Yellow box: active joint reposition error (AJRE) testing was performed in the middle 50% of the active lumbopelvic range of motion, where muscle spindles contribute most to proprioception. (**D**) The representative subject time series of a single AJRE trial without vibration. (**E**) A single trial from the same subject with a vibration. The vertical dotted line at 5 s represents the onset of a vibration stimulus.

We developed and calibrated a custom muscle vibration system using linear actuators (C2-HDLF; Engineering Acoustics, Inc., Casselberry, FL, USA; diameter: 1.2”) controlled by an audio amplifier (AK-170; CACAGOO, China). Additional details on the vibration system components can be found in the Supplementary Materials. Our system allowed us to manipulate the frequency (70–100 Hz) and amplitude (0.2–1.0 mm) of the vibration within the range of parameters used in previous studies [17,18,31,34,43]. Muscle vibrators were applied bilaterally to the lumbar erector spinae muscles two centimeters lateral to the fourth lumbar spinous process (Figure 1A). Care was taken to ensure the vibrators were applied over the muscle belly and not directly over the bone. Tegaderm and tape were used to secure the vibrators, which removed the need for a belt that wraps around the trunk. We quantified joint position using a 2D electrogoniometer (Noraxon, Scottsdale, AZ, USA, Inc., model 508) secured with double-sided tape at the spinous process of L1 and on the sacrum at the level of S2 (Figure 1A).

Each participant performed lumbopelvic active joint reposition error (AJRE) testing while blindfolded to determine the TEM vibration parameters that induced maximal altered proprioception [16,44,45]. Verbal cues were provided to elicit movements of the lumbar spine and pelvis through anterior and posterior pelvic tilting (Figure 1B). Initially, participants completed two trials that involved actively tilting their pelvis anteriorly and then posteriorly five times through their full lumbopelvic range of motion (Figure 1C). These active range-of-motion trials allowed for tissue preconditioning, and data from the second trial was used to select target lumbopelvic positions (angles) in the middle 50% of the participant’s range of motion (yellow box in Figure 1C). Muscle spindle afference is the primary contributor to proprioception in the middle ranges, as opposed to the end range, where joint and cutaneous receptors contribute additional sensory information [3,4,8].

Participants subsequently performed AJRE testing with and without TEM vibration. At the start of each trial, subjects were verbally guided to one of three lumbopelvic target positions. They then held the target position for 5 s before tilting their pelvis anteriorly and posteriorly through their full lumbopelvic range of motion two times. They then actively tilted their pelvis to match the target position and were instructed to hold at the estimated target position for 5 s (Figure 1D). There was no time limit on how long participants had to reproduce the target position.

Subjects performed 6 baseline trials of AJRE testing without vibration. We selected this number of trials based on the results of pilot testing to allow for familiarization with the task and the stabilization of performance. In addition, the final three baseline trials were used to determine baseline AJRE performance. Participants then completed AJRE trials with TEM vibrations at frequencies of 70, 80, 90, and 100 Hz with a constant peak-to-peak amplitude of 0.5 mm. Vibration frequencies were presented three times each in a pseudorandom and interleaved order with one-minute rest breaks between trials at different frequencies (Figure 1E) [17,18,31,34,43]. Within each trial, the vibration began immediately after the target position was held for 5 s (dotted vertical line in Figure 1E) and persisted until the end of each trial (after the estimated target position was held for 5 s).

Several steps were taken to control for the saturation of muscle spindle activity and for potential cumulative effects of vibration over time. First, the four vibration frequencies were presented three times, each in a pseudorandom and interleaved order. Second, vibration frequencies were tested in two blocks with a longer 5 min rest period between the two blocks. In pilot testing, we determined that 5 min following the first block of AJRE trials was sufficient to allow the error to return to being near the baseline. During this longer rest period, participants were free to move as they wanted; this allowed time for lumbopelvic movement with accurate TEM proprioceptive information. Finally, following the longer 5 min rest period and before starting the second block of vibration trials, participants performed an additional 3 trials of AJRE testing without vibration. This block served as an additional check to ensure the full washout of the previous vibration effects. A flow chart demonstrating our procedure for AJRE testing is included in the Supplementary Materials.

The target position (lumbopelvic angle) for each AJRE trial was calculated as the mean of the first 5 s of data collection (Figure 1D,E). The lumbopelvic angle associated with repositioning at the target was calculated as the mean of the final 5 s of data. Error in each AJRE trial was calculated as the difference, in degrees, between the target and reposition angles. Absolute error was then calculated as the average error in the three trials without vibration (baseline) and the three trials at each vibration frequency. The vibration frequency that induced the greatest absolute error was selected as the personalized vibration frequency for each participant in trunk postural control testing.

### 2.3. Seated Trunk Postural Control Testing

**Figure 2:**
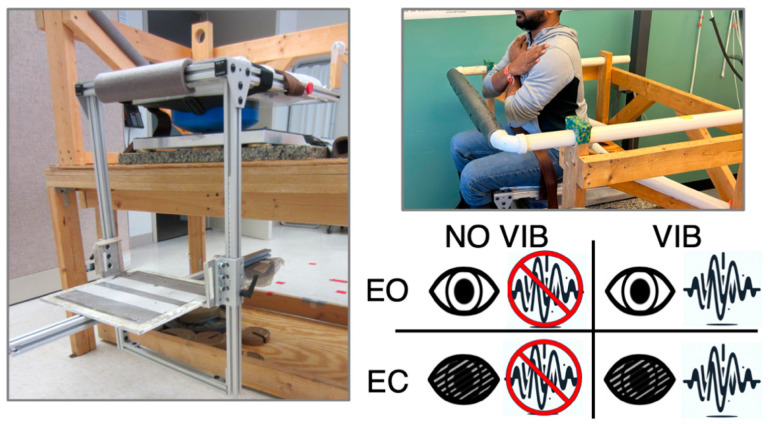
(**Left**) The seated testing apparatus with an unstable chair atop a force plate. The solid, blue, 44 cm-diameter hemisphere makes the chair unstable, and the position is adjusted to accommodate different thigh lengths. The footplate is also adjustable to allow for the standardization of the testing position. (**Top Right**) A participant in the testing position with arms crossed. A safety bar positioned in front of participants was used to steady the chair during rest periods. (**Bottom Right**) Trunk postural control was tested under four conditions: eyes open—no vibration (EO); eyes open—vibration (EO VIB); eyes closed—no vibration (EC); and eyes closed—vibration (EC VIB).

After identifying personalized vibration parameters, participants were provided a 10 min rest break to allow for the washout of vibration aftereffects and to provide the TEMs time to recover following repeated movements. This additional time allowed the tester to calculate the absolute error values used to select the personalized vibration parameters for trunk postural control testing. Participants were positioned in a seated testing apparatus developed to isolate the postural control of the trunk. The chair is made unstable by a hemisphere (44 cm diameter) being positioned under the seat (Figure 2). Additional details of the characteristics of the unstable chair are presented in the Supplementary Materials. Participants actively controlled their upright seated posture by balancing themselves for 30 s with the instruction to “balance yourself and remain as still as possible”. Trunk postural control was assessed using a force plate (Kistler, Novi, MI, USA) positioned under the hemisphere. For all tests, subjects were asked to sit with their arms crossed and hands just under the clavicles. The hips, knees, and ankles were all positioned at 90 degrees of flexion, and the feet were positioned flat on the chair’s footplate. Participants were provided practice trials in the EO and EC conditions. Participants were provided 30 s rest breaks between trials, where they could stabilize the seat by holding onto the safety bar positioned in front of them (Figure 2). Four conditions were tested: Condition 1: eyes open without vibration (EO); Condition 2: eyes open with vibration (EO VIB); Condition 3: eyes closed without vibration (EC); and Condition 4: eyes closed with vibration (EC VIB). Three trials were performed for each condition in a block-randomized order, meaning there were three blocks of four trials, with each of the four conditions presented randomly once in each block.

### 2.4. Data Processing

Postural control and AJRE testing data were processed using custom software (Labview, version 8.6; National Instruments, Austin, TX, USA). Electrogoniometer data were collected at 1200 Hz and low-pass-filtered at 5 Hz with a fourth-order Butterworth digital filter. Force plate data were collected at 1200 Hz, filtered using a piecewise linear filter, and down-sampled to 240 Hz. Center of pressure time series data were calculated. Our primary outcome measures quantifying trunk postural control performance were the area of a 95% confidence ellipse (CEA_95_) and the mean velocity (MVEL) of the center-of-pressure path, representing spatial and temporal measures of task performance, respectively. We also looked at the MVEL in the medial–lateral (MVEL_ML_) and anterior–posterior (MVEL_AP_) directions. Smaller values for all outcome variables indicated better performance.

### 2.5. Statistical Analysis

To determine the change in absolute error from the baseline to each vibration frequency, we calculated the mean within-subject effect sizes (Cohen’s d) for vibration frequencies of 70 Hz, 80 Hz, 90 Hz, and 100 Hz when applied standardly to all participants. Further, we used the same within-subject effect size measure to characterize the magnitude of the effects of personalized vibration parameters. To assess the effects of TEM vibrations on seated trunk postural control, a 2 × 2 (vibration condition × visual condition) repeated measures ANOVA in the group with no back issues was performed. Effect sizes were obtained by computing the partial eta squared (η_p_^2^) for the main effects and interactions. Effect sizes were interpreted as small (η_p_^2^ > 0.01), medium (η_p_^2^ > 0.06), and large (η_p_^2^ > 0.14) [46]. Mean within-subject effect sizes (Cohen’s d) were also calculated for the differences in seated trunk postural control between the EO and EO VIB conditions, the EO and EC conditions, and the EC and EC VIB conditions. Effect sizes were interpreted as small (d = 0.2), moderate (d = 0.5), and large (d = 0.8).

We used independent *t*-tests and chi-squared tests to investigate differences between the group with no back issues and the matched cLBP group for demographics and self-report measures. Independent t-tests were used to confirm that baseline group differences existed in seated trunk postural control between participants with no back issues without vibrations and individuals with cLBP. We utilized equivalence testing to test the hypothesis that TEM vibration would degrade the seated postural control in individuals with no back issues, resulting in performance equal to that of persons with cLBP. We calculated between-group mean difference scores and compared these to minimally detectable change (MDC_95_) scores [36]. We also calculated between-group effect sizes (Cohen’s d). We considered the group’s equivalent if the mean difference was less than the MDC_95_ and the effect sizes were very small (<0.2).

## 3. Results

### 3.1. Active Joint Reposition Error

**Figure 3:**
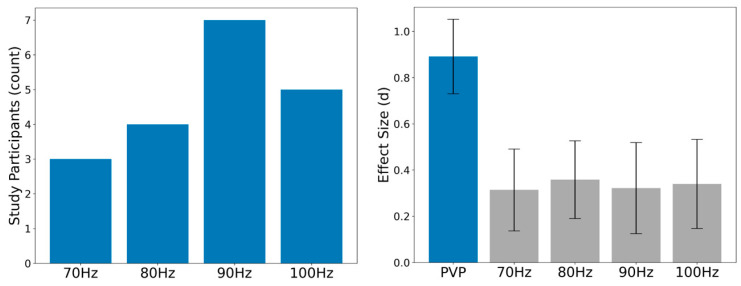
(**Left**) The number of participants whose active joint reposition absolute error was greatest at each frequency tested. (**Right**) the mean within-subject effect sizes (d) of the change in absolute error during active joint reposition error testing (relative to baseline with no vibration) when using personalized vibration parameters (PVPs), and if each participant were tested at the same standard frequency. Error bars represent standard error.

Participants in this study demonstrated between-subject variability in the vibration frequency that induced the highest absolute error in AJRE testing (Figure 3, Left). The frequency that induced the highest absolute error in the greatest number of participants was 90 Hz (n = 7), but 80 and 100 Hz each induced the highest absolute error for roughly a quarter of participants (n = 4 and n = 5, respectively). Participants exhibited small mean within-subject effects (d = 0.31–0.36) for increases in the absolute error from baseline when all participants were given the same frequency. Personalized vibration parameters induced a large mean absolute error within-subject effect size (d = 0.89) (Figure 3, Right). Mean absolute error scores for all testing conditions are presented in the Supplementary Materials.

### 3.2. Seated Trunk Postural Control

**Figure 4:**
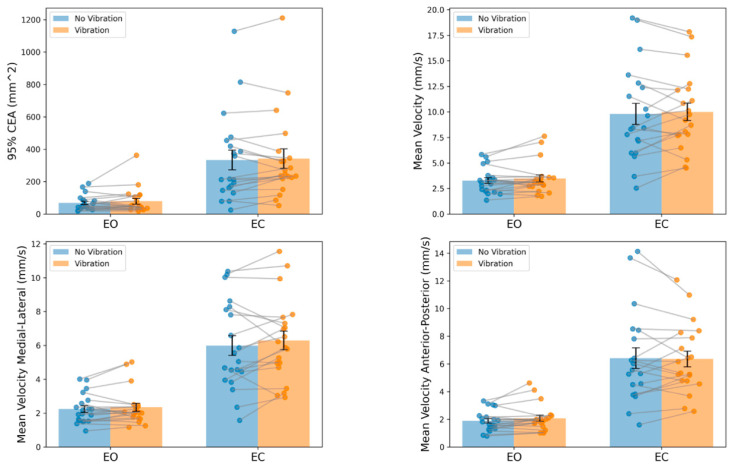
Results for seated trunk postural control testing in the group with no back issues. Bars represent group means, error bars represent standard error. Dots represent individual subjects and lines connect the eyes-open (EO) and eyes-closed (EC) conditions for the same person. Significant main effects for vision were observed where the 95% confidence ellipse area (CEA), mean velocity, and the mean velocity in the sagittal and frontal planes were increased in the eyes-closed condition (*p* < 0.001). No significant effects of vibration were detected for any postural control measure. Note the heterogeneity in response to vibration where some participants improved (a negative sloped line) and others had poorer performance with vibration (a positive sloped lines).

Results for repeated measures ANOVA testing in the group with no back issues are presented in Figure 4. The ANOVA for CEA_95_ revealed significant main effects for vision (F = 29.0; *p* < *0*.001; η_p_^2^ = 0.604) but not for vibration (F = 1.4; *p* = 0.247; η_p_^2^ = 0.070). For the MVEL, large effects were observed for vision (F = 96.6; *p* < 0.001; η_p_^2^ = 0.836), but no main effect for vibration (F = 1.0; *p* = 0.320; η_p_^2^ = 0.052) was found. Findings were similar when breaking down the MVEL into component directions. Large significant main effects for vision were present for both the MVEL_ML_ (F = 100.9; *p* < 0.001; η_p_^2^ = 0.842) and MVEL_AP_ (F = 29.0; *p* < 0.001; η_p_^2^ = 0.604), and neither the MVEL_ML_ (F = 1.81; *p* = 0.185; η_p_^2^ = 0.091) nor the MVEL_AP_ (F = 0.2; *p* = 0.648; η_p_^2^ = 0.011) exhibited effects for vibration. There were no significant interaction terms for any measure (*p* ≥ *0*.423).

In addition to repeated measures ANOVA testing, we calculated within-group effect sizes (Cohen’s d) relative to the EO condition to characterize the magnitude of effects when impairing proprioception, vision, and proprioception and vision simultaneously. We also calculated the effect size between EC and EC VIB conditions to elucidate the effects of vibration in the absence of visual feedback (Table 2). Effect sizes were consistent across measures and conditions where the effects of vibration were small or trivial in the EO (EO VIB—EO, d = 0.21–0.37) and EC (EC VIB—EC, d = −0.04–0.27) conditions. The effects of eliminating vision alone were large (EC-EO, d = 1.14–1.94). Means and standard deviations for the CEA_95_, MVEL, MVEL_ML_, and MVEL_AP_ for each condition are included in the Supplementary Materials.

### 3.3. Comparison with cLBP

We observed baseline group differences in postural control performance between participants with no back issues without vibrations and individuals with cLBP in the EO condition for the CEA_95_ (*p* = 0.048), MVEL (*p* = 0.023), MVEL_ML_ (*p* = 0.027), and MVEL_AP_ (*p* = 0.022). In the EC condition, differences were discovered for the MVEL_ML_ (*p* = 0.037), and trends were detected for the CEA_95_ (*p* = 0.068) and MVEL (*p* = 0.065).

To determine if the TEM vibration in people with no back issues induced changes in trunk postural control equivalent to the impaired performance in people with cLBP, we evaluated mean difference scores and effect sizes between groups. For each measure of postural control, in both EO and EC conditions, we observed mean differences that were less than the MDC_95_. However, all effect sizes were d > 0.2 in both visual conditions; thus, all exceeded our a priori threshold for performance equivalence (Table 3).

## 4. Discussion

This study aimed to understand the effects of vibration-induced altered TEM proprioception on trunk postural control. We hypothesized that personalizing vibration parameters would result in greater errors in proprioception than a single vibration frequency applied to all participants. Our results support this hypothesis. We hypothesized that healthy individuals would demonstrate poorer postural control performance while undergoing TEM vibration compared to no-vibration conditions, but we did not observe an effect of vibration on any measure of trunk postural control. We also explored the relationship between trunk postural control in adults with no back pain with vibration-induced altered proprioception and age- and sex-matched adults with cLBP. We hypothesized that trunk postural control performance would be equivalent between healthy individuals experiencing TEM vibration and individuals with cLBP. While mean differences were less than the MDC_95_, effect sizes exceeded our a priori threshold of 0.2. This indicates that adults with cLBP have impaired trunk postural control but fails to support our hypothesis that altered TEM proprioception explains the postural control performance degradation seen in individuals with cLBP.

To our knowledge, this is the first study to determine and employ personalized vibration parameters to optimally alter TEM proprioception. We provide evidence supporting between-subject variability in the vibration frequency that maximally induces proprioceptive impairment. Additionally, our results indicate that using a single vibration frequency for all study participants results in a small effect, whereas personalizing the vibration frequency results in a large effect on lumbopelvic repositioning accuracy. Findings suggest that research designs that choose to implement a single set of vibration parameters for all participants are leaving potential effects unrealized. We found robust effects of TEM vibrationw on proprioception during lumbopelvic AJRE testing. This replicates a previous study that found that TEM vibration impaired lumbopelvic active joint reposition sense in sitting in adults with no back pain [32]. The lumbopelvic active range of motion (11–47°) and reposition error values (0.2–6.0°) recorded for this study were similar to those of previous studies in the same population [32,44]. In 15/19 participants, differences in the mean absolute error between baseline and personalized vibration parameters exceed the standard error of the measurement (0.40°) established for a similar lumbopelvic AJRE testing protocol [44].

Despite the robust effects of trunk extensor muscle vibration on proprioception during AJRE testing and the amplification of vibration effects with personalization, we observed trivial-to-small effects of vibrations on trunk postural control during unstable sitting both in EO and EC conditions. There are several reasons why this might be the case. Seated postural control on an unstable surface requires the sensory integration of multiple information streams, including visual, vestibular, and somatosensory signals. Trunk muscle spindle afference is only one type of somatosensory signal, and the lumbar erector spinae muscles are but one group of the many trunk muscles whose spindle afference provides information pertinent to trunk control in unstable sitting. We believe the redundancy present in sensory information streams and trunk musculature makes the unstable sitting task less sensitive to changes that are limited to TEM proprioception. AJRE testing, on the other hand, requires the cognitive evaluation of the specific position of the lumbar spine and pelvis, the maintenance of that position in memory, and recreating the target position through movement that changes TEM length and tension.

Another potential reason we did not detect changes in seated postural control during vibration conditions is the observed between-subject heterogeneity in response to vibrations. Some participants improved postural control performance with the addition of vibrations, others had poorer postural control, and some demonstrated virtually no change (Figure 4). These divergent effects resulted in the inability to detect a main effect of vibration. We looked for patterns in the response to vibrations among our study participants. For the EC condition, six participants demonstrated increases in the MVEL with vibration that exceeded the MDC_95_; alternatively, five participants exhibited decreases in the MVEL that reached the MDC_95_ threshold. For the remaining nine participants, we cannot confidently say that vibration impacted their performance on this measure as changes with vibration did not exceed the MDC_95_ threshold. Interestingly, heterogeneity in response to vibration has been described previously in the cLBP literature. During a precision trunk control task, individuals with cLBP demonstrated non-significant reductions in tracking error with TEM muscle vibration, whereas healthy controls exhibited a significant increase in tracking error [47]. A similar finding was also reported for a trunk force reproduction accuracy task [31]. Individuals with cLBP demonstrated a significant reduction in force matching errors during TEM vibration, whereas participants without cLBP showed a significant increase in errors with vibration [31,48]. A consistent finding of improvement in trunk sensorimotor control with TEM vibration in cLBP makes the participants in this study, those without back pain, who performed better with vibration a potentially interesting subgroup to follow longitudinally, especially considering that altered trunk control has been associated with the development of low back pain [5]. Additionally, future studies should investigate the potential of TEM vibration to enhance motor control in individuals with cLBP. 

This paper adds to the literature that consistently describes the importance of vision in trunk postural control [34,49]. Previous studies have documented that moving from having EO to having EC induces large changes in postural control, often much larger than other methods of impairing sensory systems such as vibration [34] or unstable surfaces [49]. Willigenburg et al. [34] also used vibrations on TEMs while evaluating postural control in unstable sitting. Similar to the current study, no effects of vibration on sway amplitude (the root-mean-square of the center-of-pressure with respect to its mean) were found. However, in contrast to the current study, a statistically significant 12% increase in the MVEL was reported when vibration was applied with the participant’s EO. Comparatively, an EC condition without vibration resulted in significant increases in sway amplitude in multiple directions, and the MVEL approximately doubled from the condition with EO and no vibration [34].

We found baseline group differences in postural control between healthy individuals and demographically similar people with cLBP. This replicates findings from previous studies using unstable sitting paradigms [36,49,50]. The effects of vibration-induced altered TEM proprioception did not result in equivalent trunk control impairment as measured in our cLBP sample. This is not surprising considering the negligible effects of vibration on trunk postural control in the group with no back issues.

Chronic back pain is a multi-faceted neuromusculoskeletal condition. We hypothesized that manipulating TEM spindle afference would be enough to induce the trunk postural control changes observed in individuals with chronic back pain. We were incorrect. In hindsight, this is not surprising, considering individuals with cLBP may have other impairments that impact performance in addition to impaired trunk proprioception. The pain present in the cLBP group may influence the somatosensory feedback and could influence results [51]. Muscle coordination and activation timing are also different in individuals with cLBP [52,53,54,55]. Additionally, chronic low back pain is known to exhibit alterations at multiple levels of the central nervous system [56,57,58,59,60] and in musculotendinous tissue [6]. Mimicking the effects of cLBP on trunk postural control may require manipulating more than just TEM proprioception. Rehabilitation strategies aiming to improve trunk postural control for individuals with cLBP should consider the multitude of factors that influence trunk postural control and not focus exclusively on trunk extensor muscle proprioception.

A limitation of this study, and another potential reason that the effects of TEM vibration on postural control were negligible, is that we only applied vibrations to the lumbar extensor muscles. Future studies should investigate applying vibrations to multiple groups of trunk muscles. While presenting four vibration frequencies improves the current status quo in muscle vibration studies, future work could include more vibration frequencies and different amplitudes to explore these parameters further. We did not investigate the effects of vibrations on trunk postural control in standing. While assessments in standing limit the conclusions that can be drawn specifically about the TEM spindle function, standing remains a functionally relevant posture. Future studies should assess postural control in both sitting and standing to evaluate similarities and differences in trunk control relative to different postures. Finally, other paradigms may better model the impaired seated trunk postural control observed in individuals with cLBP. For example, cooling or anesthetizing the TEM may alter the seated trunk postural control in individuals with no back issues and may result in an alteration that is more like that observed in individuals with cLBP. These and other possible paradigms were not investigated in the current study.

## 5. Conclusions

Vibration alters position sense during active joint reposition error testing, and personalizing vibration parameters amplifies the size of this effect. However, we measured trivial-to-small effects of trunk extensor muscle vibration on seated trunk postural control in adults with no back pain, which did not result in performance degradation equivalent to that observed in individuals with cLBP.

## Figures and Tables

**Table 1:** Participant demographics and self-report measures.

	HC (*n* = 20)	cLBP (*n* = 20)	*p*-Value
Sex	10 F/10 M	10 F/10 M	1.00
Age (years)	28 (9)	29 (9)	0.98
Body Mass Index (BMI)	24.5 (2.3)	26.2 (4.4)	0.12
Modified Baecke PA (0–15)	9.6 (1.3)	9.3 (1.7)	0.52
Pain: 7-day avg (0–10)	NA	4.4 (1.6)	NA
Oswestry Disability Index (0–50)	NA	7.7 (3.2)	NA

Data presented as means (SD). *p*-values are associated with chi-square or *t*-tests for group differences. HC: healthy control; cLBP: chronic low back pain; PA: physical activity.

**Table 2:** The within-group mean difference scores and effect sizes for seated trunk postural control in participants with no back issues across conditions.

	CEA_95_ (mm^2^)		MVEL (mm/s)		MVEL_ML_ (mm/s)		MVEL_AP_ (mm/s)	
	M (SD)	ES (d)	M (SD)	ES (d)	M (SD)	ES (d)	M (SD)	ES (d)
EO VIB—EO	9.8 (46.2)	0.21	0.2 (0.7)	0.28	0.1 (0.5)	0.23	0.2 (0.5)	0.37
EC—EO	265.1 (233.1)	1.14	6.5 (3.5)	1.84	3.7 (1.9)	1.94	4.5 (2.7)	1.67
EC VIB—EC	8.7 (62.5)	0.14	0.2 (1.7)	0.12	0.3 (1.1)	0.27	−0.1 (1.3)	−0.04

CEA_95_: the 95% confidence ellipse area; MVEL: the mean velocity; MVEL_ML_: the mean velocity in the medial–lateral direction; MVEL_AP_: the mean velocity in the anterior–posterior direction. EO: eyes open; VIB: vibration; EC: eyes closed. M: mean difference; ES (d): within-subject effect size calculated as the mean difference/SDD where SDD = the standard deviation of the differences.

**Table 3:** The effects of TEM vibration in healthy controls compared with chronic low back pain.

	cLBP	HC VIB	Mean Diff.	MDC_95_	ES (d)
	Eyes Open				
CEA_95_ (mm^2^)	103.5 (78.3)	78.3 (80.1)	25.2 (25.7)	154.6	0.32
MVEL (mm/s)	4.6 (2.6)	3.5 (1.6)	1.1 (0.7)	1.6	0.51
MVEL_ML_ (mm/s)	3.0 (1.5)	2.3 (1.1)	0.7 (0.4)	1.8	0.50
MVEL_AP_ (mm/s)	2.8 (1.8)	2.1 (1.0)	0.7 (0.5)	1.6	0.49
	Eyes Closed				
CEA_95_ (mm^2^)	469.6 (291.7)	342.2 (267.1)	127.4 (90.7)	440.8	0.45
MVEL (mm/s)	12.4 (6.0)	10.0 (3.8)	2.4 (1.6)	4.2	0.47
MVEL_ML_ (mm/s)	7.8 (3.4)	6.3 (2.4)	1.5 (1.0)	2.6	0.48
MVEL_AP_ (mm/s)	8.0 (4.4)	6.4 (2.5)	1.7 (1.2)	2.8	0.45

Data presented as means (SD). cLBP: chronic low back pain; HC VIB: control participants with no back issues with vibration; ES: the effect size, where (mean cLBP—mean HC VIB)/pooled SD. CEA_95_: the 95% confidence ellipse area; MVEL: the mean velocity; MVEL_ML_: the mean velocity in the medial–lateral direction; MVEL_AP_: the mean velocity in the anterior–posterior direction.

## Data Availability

The authors will make the raw data supporting this article’s conclusions available upon reasonable request.

## Supplementary Materials

The following supporting information can be downloaded at: https://www.mdpi.com/article/10.3390/brainsci14070657/s1, S1: Supplemental Materials.
