# Supplementary material for: Vibration-Induced Alteration in Trunk Extensor Muscle Proprioception as a Model for Impaired Trunk Control in Low Back Pain
Source: Brain Sci. 2024 Jun 28;14(7):657. doi: 10.3390/brainsci14070657 (PMC11274553; doi:10.3390/brainsci14070657)
Supplement: Supplementary file 1 [file brainsci-14-00657-s001.zip › brainsci-3073033-supplementary.pdf]

Supplemental Materials

Detailed Inclusion/Exclusion Criteria

Study Inclusion Criteria
Adults between the ages of 18-65 years old, fluent in English-language (self-report measures in English). This age range ensures skeletal maturity with a reasonably low prevalence of severe osteoarthritis or osteopenia.

1. **Healthy Control Group**
    a. Inclusion criteria: must self-report no low back pain for which they have sought medical attention or resulted in greater than 3 days of significant loss of ability to accomplish daily function within the last 5 years.
    b. Exclusion criteria: a history of concussion or migraine headaches within the last six months, a history of neurological disease, previous spine or hip surgery, a diagnosis of fibromyalgia, rheumatoid arthritis, or chronic fatigue syndrome, or were currently taking opioids

2. **Chronic Low Back Pain Group:** The individuals with this group were age (+/- 5 years) and sex-matched to the back-healthy group.
    a. Inclusion criteria are consistent with the Chronic Low Back Pain Minimal Dataset (Deyo et al., 2014) and include: (1) **duration of the current episode of low back symptoms greater than 3 months;** (2) back pain impacting function at least ½ days in last 3-6 months, and (3) ability to identify activities that they were currently unable to perform without pain or limitation. Participant characteristics also include age ranges of 18-65 years old.
    b. Exclusion criteria: Additional exclusion criteria include the presence of any of the following conditions as determined by prior medical and/or radiographic examination or initial MRI:
        - spinal or hip osteoporosis;
        - inflammatory joint disease;
        - any current (within 5 years) neoplastic condition;
        - any history of a vertebral fracture with current bony instability or measurable deformity;
        - severe lumbar stenosis (defined as an A-P diameter of the thecal sac of less than 5 mm at any level, from mid-sagittal lumbar T_2-weighted MRI);
        - any abnormalities or compression of the spinal cord or cauda equina;

- compression of a spinal nerve with accompanying clinical symptoms that demonstrate significant loss of or absence of sensation or muscle weakness;
- any lower extremity peripheral nerve impairment;
- injury to your legs that significantly limits your hip or knee motion;
- unstable angina, congestive heart failure, orthopnea, or severe hypertension;
- any history of a surgical procedure to the lumbar spine;
- any surgical procedures to the abdomen, thorax, upper extremities, head or neck in the 6 months prior to enrollment in the study;
- current use of any of the following medications: prescribed anticoagulants (this does not include low doses of ASA or NSAIDs), and oral or injected corticosteroids;
- current use of narcotic medication (opioids) for your back pain that the patient is unable or unwilling to abstain from for 48 hours,
- severe obesity may be an exclusion factor if it precludes the participant from fitting in the 24" bore of the MRI scanner. The usual weight limit is approximately 350 lbs.
- Currently involved in an unsettled worker's compensation claim or in personal injury litigation.
- Currently applying for, permanent or temporary disability due to a medical or mental health condition, unsettled case.
- Activity-limiting pain arising from any site other than listed in the specific entry criteria.
- The presence of any conditions that would contraindicate MRI.

Vibration System

A custom vibration system was developed using linear actuators (C2-HDLF; Engineering Acoustics, Inc., Casselberry, FL, USA) (A) and an audio amplifier (AK-170; CACAGOO, China) (B).

Linear actuators (A) have diameter of 1.2” and a weight of 30g.

A) 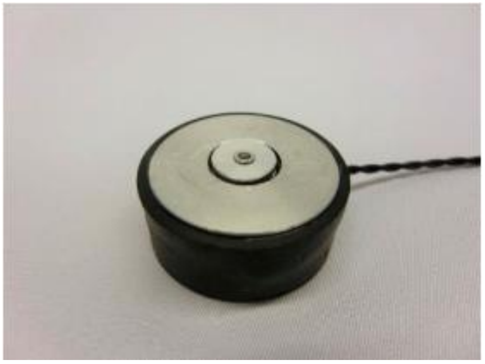

B) 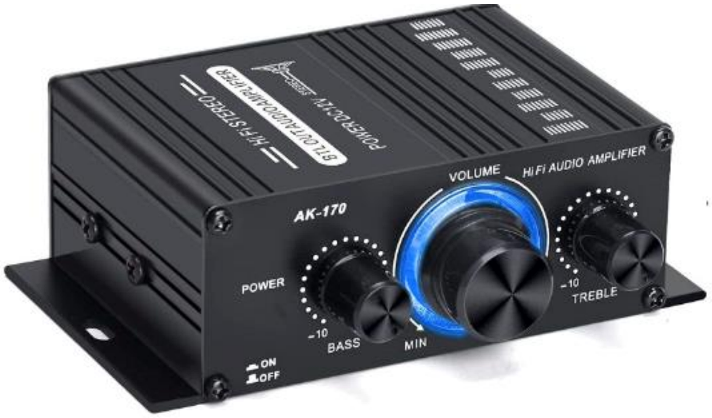

Flow Chart of Active Joint Reposition Error Testing

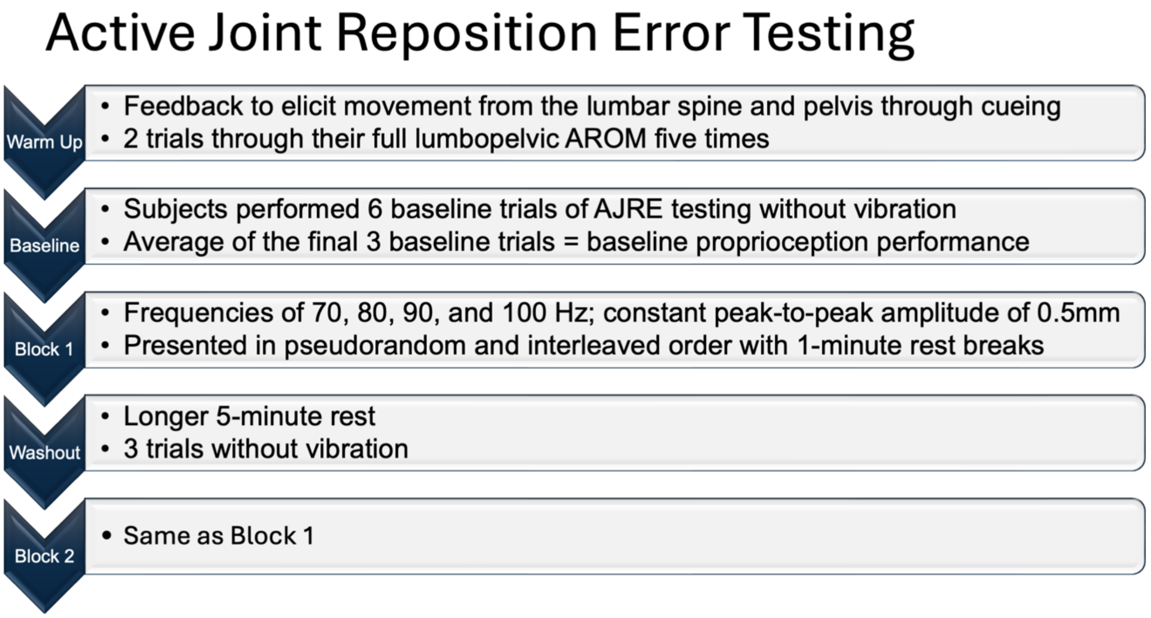

Balance Chair Specifications

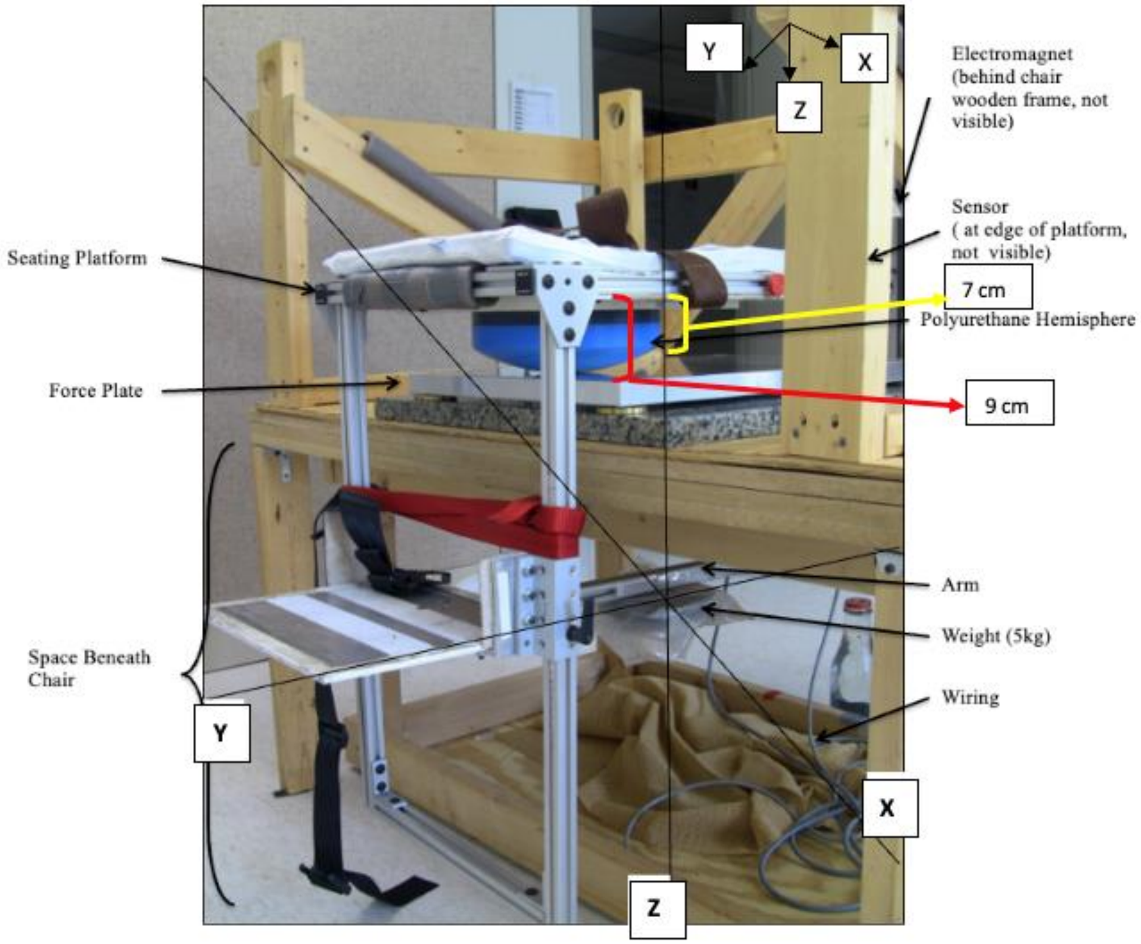

44 cm diameter hemisphere- which allows approximately 15° of tilt of the chair in any direction without the chair frame hitting the force plate; curved surface of hemisphere starts 7 cm below the seat surface with pivot point is 9 cm under the seat; with addition of the sliding plate, and the t-form (inside the white pillowcase) on top of it, the pivot point of the hemisphere is roughly 12 cm below the point of subject/ seat contact.

To balance the system (chair and subject), the location of the hemisphere along the y-direction can be changed, as a plate within the visible seat frame can slide front to back.

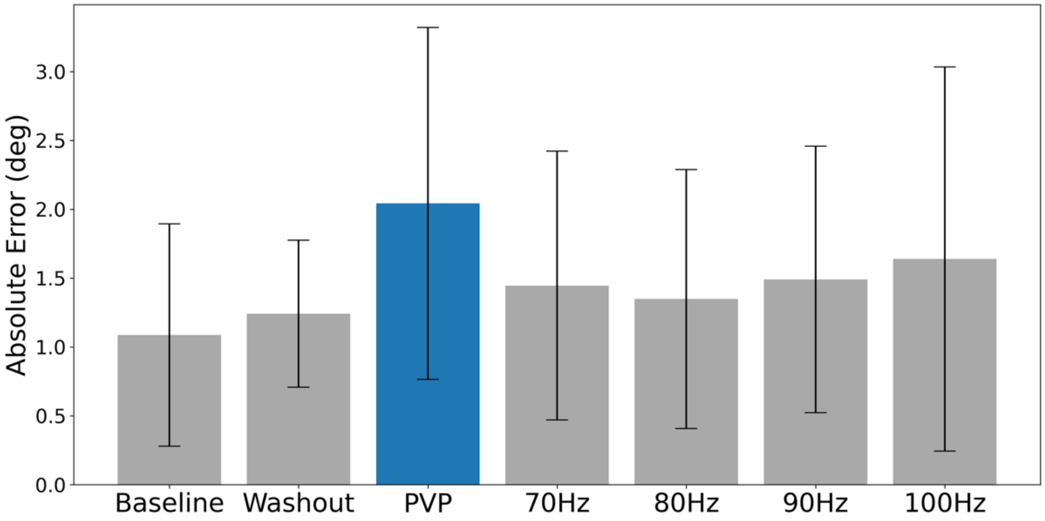

Supplemental Figure. Mean absolute error across testing conditions. Error bars represent standard deviation.

Supplemental Table. Means and Standard Deviations for Seated Trunk Control Performance in Healthy Controls Across Conditions

	EO	EO VIB	EC	EC VIB
	M (SD)	M (SD)	M (SD)	M (SD)
CEA_{95} (mm^2)	68.4 (48.0)	78.3 (80.1)	333.5 (272.7)	342.2 (267.1)
MVEL (mm/s)	3.3 (1.3)	3.5 (1.6)	9.8 (4.6)	10.0 (3.8)
$MVEL_{ML}$ (mm/s)	2.2 (0.9)	2.3 (1.1)	6.0 (2.6)	6.3 (2.4)
$MVEL_{AP}$ (mm/s)	1.9 (0.8)	1.2 (1.0)	6.4 (3.3)	6.4 (2.5)

EO: eyes open; VIB: vibration; EC: eyes closed. M: mean; SD: standard deviation; CEA_{95}: 95% confidence ellipse area; MVEL: mean velocity; $MVEL_{ML}$: mean velocity in medial-lateral direction; $MVEL_{AP}$: mean velocity in anterior-posterior direction.

Absolute vs Content Error

We used the mean absolute error to quantify proprioceptive alteration during AJRE testing. Absolute error does not provide information on the direction of the error, whereas a measure like content error does. We made the decision a priori to use mean absolute error for determining personalized vibration parameters. We explored, post hoc, the content error results to determine if these data are useful in explaining the heterogeneity of response to vibration during postural control testing.

In 15/19 (79%) participants, the frequency that induced the highest mean absolute error was the same frequency that induced the highest mean content error. The remaining four participants had mean content error values that were in the same direction but slightly smaller than their mean absolute errors. Errors were in the direction of greater posterior pelvic tilt, which aligns with an illusion of lengthened trunk extensor muscles. Seated postural control performance changes induced by vibration in the four participants whose highest mean content error and highest mean absolute error did not occur at the same vibration frequency did not follow a consistent pattern. Some of these participants improved with vibration, while others performed worse, and this varied sometimes even within subjects based on the conditions. Future investigations may consider content error or multiple sources of information regarding error to establish personalized vibration parameters.
